# Collagen Can Selectively Trigger a Platelet Secretory Phenotype via Glycoprotein VI

**DOI:** 10.1371/journal.pone.0104712

**Published:** 2014-08-12

**Authors:** Véronique Ollivier, Varouna Syvannarath, Angèle Gros, Amena Butt, Stéphane Loyau, Martine Jandrot-Perrus, Benoît Ho-Tin-Noé

**Affiliations:** 1 Inserm Unit 1148, Laboratory for Vascular Translational Science, Paris, France; 2 Paris Diderot University, Paris, France; 3 Paris 13 University, Villetaneuse, France; 4 Assistance Publique-Hôpitaux de Paris, Hôpital Bichat Claude Bernard, Paris, France; King’s College London School of Medicine, United Kingdom

## Abstract

Platelets are not only central actors of hemostasis and thrombosis but also of other processes including inflammation, angiogenesis, and tissue regeneration. Accumulating evidence indicates that these “non classical” functions of platelets do not necessarily rely on their well-known ability to form thrombi upon activation. This suggests the existence of non-thrombotic alternative states of platelets activation. We investigated this possibility through dose-response analysis of thrombin- and collagen-induced changes in platelet phenotype, with regards to morphological and functional markers of platelet activation including shape change, aggregation, P-selectin and phosphatidylserine surface expression, integrin activation, and release of soluble factors. We show that collagen at low dose (0.25 µg/mL) selectively triggers a platelet secretory phenotype characterized by the release of dense- and alpha granule-derived soluble factors without causing any of the other major platelet changes that usually accompany thrombus formation. Using a blocking antibody to glycoprotein VI (GPVI), we further show that this response is mediated by GPVI. Taken together, our results show that platelet activation goes beyond the mechanisms leading to platelet aggregation and also includes alternative platelet phenotypes that might contribute to their thrombus-independent functions.

## Introduction

Platelets are most commonly known for their central role in hemostasis and thrombosis, both of which rely on the so-called mechanism of platelet activation. More precisely, current knowledge of platelet activation describes it as the transition from a functionally resting state to a procoagulant and prothrombotic platelet phenotype. This multistep process is initially evoked by interactions of platelets with adhesive components of the subendothelial extracellular matrix at sites of vascular injury or by soluble platelet agonists. Such stimulated platelets go through morphological changes but above all, they undergo functional changes, with activation and surface expression of integrins and other adhesion molecules, exposure of procoagulant phosphatidylserine, and secretion of thrombogenic substances from their storage granules. Altogether, these changes result in the formation of either the primary hemostatic plug or a pathologic thrombus [1]–[3]. This pattern of platelet activation has been extensively studied and is now widely accepted as the mechanism supporting platelet contribution to primary hemostasis and thrombosis. For this reason, when talking about platelet activation, one usually refers to this stereotypic adhesive, procoagulant and prothrombotic platelet state. Nevertheless, evidence that platelets can present various levels of activation has been clearly provided by *in vitro* and *in vivo* experiments showing that the acquisition of their aggregative function is a sequential and gradual process, with reversible and irreversible steps [4]–[7]. The concept of differential platelet activation is further supported by recent results showing that hemostatic plugs are heterogeneous in composition, with regional differences in the extent of platelet activation [8]. Moreover, it has been shown *in vitro* that platelets can differentially release cytokines [9] and angiogenic factors [10]–[12] in an agonist dependent-manner.

The relevance of alternative states of platelet activation becomes very likely if one considers that currently platelets are not only recognized as central actors of hemostasis and thrombosis, but also as regulators of many other pathophysiological processes including innate and adaptive immune responses [13], [14], angiogenesis [15], [16], or wound healing [17]. Although the exact mechanisms underlying these “non classical” functions of platelets have not been fully elucidated, there is substantial evidence that they do not necessarily rely on the classically described activation state of platelets. For example, platelets have been shown to exert a vasculoprotective action in various inflamed organs including the skin, lungs, kidneys, and solid tumors, and this, before any signs of thrombosis are evident [18]–[21]. The fact that thrombus formation is dispensable for this beneficial action of platelets suggests the existence of alternative states of platelet activation that might be uncoupled from their procoagulant and/or prothrombotic activities. Here, we investigated this possibility through dose-response analysis of thrombin- and collagen-induced changes in platelet phenotype, with regards to morphological and functional markers of platelet activation including shape change, aggregation, integrin activation, P-selectin surface expression, and secretion of soluble compounds. Our results show that collagen at low dose triggers a non-thrombotic platelet secretory phenotype characterized by the release of various soluble platelet factors in the absence of the classical activation-associated changes.

## Methods

### Ethics Statement

All blood donors were volunteers who gave their free and informed written consent to this research study, which conforms to the ethical standards of the Declaration of Helsinki. Legal and ethical authorization for research use of collected blood was obtained through a national convention between the French National Institute of Health and Medical Research (Inserm) and the French Blood Institute (EFS) (convention number I/DAJ/C2675).

### Washed platelet preparation and stimulation

Blood from healthy volunteers who had taken no medication during the previous two weeks, was drawn into 15% (v/v) trisodium citrate acid–citric–dextrose (ACD-A, Vacutainer system; Beckton Dickinson, Le Pont-de-Clais, France). Washed platelets were prepared from isolated platelet rich plasma as previously reported [22] and resuspended at a final concentration of 2.10^8^/mL in platelet reaction buffer (Hepes 5 mM, NaHCO_3_ 12 mM, NaCl 137 mM, KCl 2 mM, CaCl_2_ 2 mM, NaH_2_PO_4_ 0.3 mM, MgCl_2_ 1 mM, glucose 5.5 mM, pH 7.4). At the end of the platelet preparation and before adding the agonists, platelets were allowed to recover from PGE1 and apyrase treatments for 20 min at 37°C. Washed platelets were then incubated with increasing doses of thrombin (purified human α-thrombin, 0.1 NIH unit/nM [23]) or fibrillar collagen from equine tendon (Horm collagen, Nycomed, Munich, Germany), either in static or stirring (500 rpm) conditions for 15 min at 37°C. In an alternative set of experiments, platelets (400 µL at 2.10^8^/mL) were stimulated in Millicell culture inserts (0.4 µm pore size, polycarbonate membrane, Millipore, Billerica, MA, USA) placed into 24-well plates containing 800 µL of reaction buffer, and incubated for 30 min at 37°C on an orbital shaker.

### Analysis of platelet morphology and P-selectin immunostaining

At the end of the incubation, 50 µL of resting or stimulated washed platelets were fixed in 950 µL of 3.7% paraformaldehyde (PFA) and centrifuged onto glass slides (15 min, 1200 g) for analysis by differential interference contrast (DIC) microscopy. For P-selectin immunostaining, platelets were permeabilized on ice with 0.1% Triton X-100 in 0.1 M citrate buffer, pH 6.0 and subjected to saturation with PBS at 3% bovine serum albumin (BSA), prior to incubation with a mouse monoclonal antibody to human P-selectin (BD Biosciences, San Jose, CA) at 5 µg/mL in PBS 1% BSA and 0.1% Tween 20. An Alexa Fluor 488-conjugated rabbit polyclonal antibody to mouse IgG (Life Technologies, Saint Aubin, France) at 2 µg/mL was used as a secondary antibody. The slides were mounted with fluorescence mounting medium (Dako, Carpinteria, CA). Observation and acquisition of DIC and fluorescence microscopy images were made using a Zeiss Axio Observer microscope (Carl Zeiss, Le Pecq, France).

### Flow Cytometry

Resting and stimulated platelets (5 µL) were incubated with a FITC-conjugated anti-P-selectin antibody or corresponding isotype-matched control (Beckman Coulter Immunotech, Marseille, France) in a final volume of 50 µL PBS. Integrin activation was assessed in a similar manner using the FITC-conjugated PAC1 antibody to activated glycoprotein IIb/IIIa. Phosphatidylserine exposure was quantified by annexin V labeling using Cy5-coupled annexin V (BD Pharmingen, San Diego, CA). A cell sample to which 5 mM EDTA was added just prior to staining with Cy5-coupled annexin V was used as a negative control. Samples were analyzed by flow cytometry using a LSRII apparatus (BD Biosciences, Le Pont de Claix, France).

For measurement of soluble PF4 directly in platelet suspensions, we used a fluorescent microsphere*-*based flow cytometric immunoassay. Briefly, COOH-functionalized green fluorescent microspheres (Estapor F1XC-200, 2.45 µm diameter, Millipore) were coated with a mouse anti-human PF4 antibody (9 µg/cm^2^ bead surface area, R&D systems, Minneapolis, MN) according to the manufacturer’s instructions. At the end of the incubation of stirred platelets, microspheres were added to 50 µL platelet suspension aliquots at a final concentration of 20 000 microspheres/µL, together with biotinylated goat IgG to human PF4 (R&D systems) and Alexa Fluor 647 streptavidin (Life Technologies). Microspheres in platelet suspensions were identified in flow cytometry by their green fluorescence and light-scattering characteristics, and their mean Alexa 647 fluorescence intensity was measured. Mean Alexa 647 fluorescence intensities were then converted into PF4 concentration according to a standard curve determined by incubating microspheres with known amounts of recombinant human PF4 (R&D systems).

### Platelet aggregation

Platelet aggregation was measured by turbidimetry at 37°C under stirring conditions in the absence of exogenous fibrinogen. Washed platelets (2.10^8^/mL) in platelet reaction buffer were activated by addition of the agonists and aggregation was followed for 8 min using an APACT-4004 aggregometer (LABiTec, Ahrensburg, Germany).

### Calcium Signaling

Washed platelets (3.10^8^/mL) in 5 mM Hepes, 137 mM NaCl, 2 mM KCl, 0.3 mM NaH_2_PO_4_, 1 mM MgCl_2_, 5.5 mM glucose, pH 7.4 (assay buffer) were incubated at 37°C for 30 min with 5 µM Oregon Green 488 BAPTA-1AM (Life Technologies, St Aubin, France). ACD-A (1/10 vol), apyrase (0.30 U/mL), PGE-1 (0.1 µM) and five volumes of wash buffer (36 mM citric acid, 5 mM glucose, 5 mM KCl, 1 mM MgCl_2_, 103 mM NaCl, 0.3 U/mL apyrase, 0.1 µM PGE-1, 0.1% BSA, pH 6.5) were then added to platelets before a 12 min centrifugation step at 1200 g. Platelets were resuspended at 2.10^8^/mL in assay buffer supplemented with 2 mM CaCl_2,_ and distributed in 96-well flat-bottomed plates. Basal fluorescence of Oregon Green 488 BAPTA-1AM-loaded platelets was followed for 2 min and calcium-dependent changes in fluorescence intensity were then recorded for 5 min after adding the agonists using a 96-well plate fluorimeter (Fluoroskan Ascent FL, Thermo Labsystems, Dreieich, Germany).

### ELISA analysis of platelet releasates

At the end of the incubation of platelets with or without the agonists and after taking platelet samples for analysis in flow cytometry and microscopy, supernatants of the remaining platelets were collected after two centrifugation steps: 12 min, 1200 g at RT and 3 min, 13000 g at RT. Aliquots of supernatants were kept frozen at −80°C until analysis. The following ELISAs were used in accordance with the manufacturer’s instructions: serotonin (GenWay, San Diego, CA, USA), platelet factor 4 (PF4) (RayBiotech, Norcross, GA, USA), TGF-β1 (Enzo Life Science, Villeurbanne, France) and angiopoietin-1 (Abcam, Paris, France).

### Measurement of ATP levels

ATP levels were measured by chemiluminescence using the ATPLite Assay System (PerkinElmer, Boston, MA). ATPLite reagent was added directly to platelet suspensions or supernatants at a 1∶10 dilution and luminescence was read using a SpectraFluor Plus plate reader (Tecan Austria Gmbh, Grodïg, Austria).

### Statistics

Data are expressed as means ± standard error of the mean (SEM) and were compared by the non-parametric Mann-Whitney test using Kaleidagraph software (Synergy Software, Reading, PA). P-values<0.05 were regarded as statistically significant.

## Results

### Dose-response analysis of thrombin- and collagen-induced platelet aggregation and shape change

**Figure 1:**
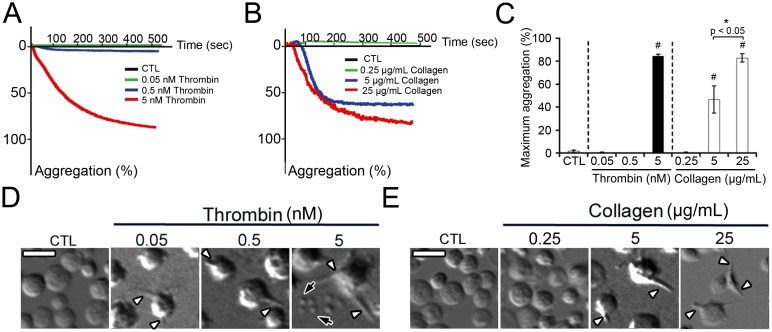
Dose-response analysis of thrombin- and collagen-induced platelet aggregation and shape change. The aggregation of human washed platelets in response to various concentrations of thrombin or collagen was analyzed by turbidimetry under stirring conditions in the absence of exogenous fibrinogen. In parallel, the individual morphological changes induced by the same concentrations of agonists were analyzed by DIC microscopy. **A–B**. Diagrams showing representative dose-response aggregation tracings obtained for thrombin (**A**) and collagen (**B**). CTL corresponds to non-treated resting platelets. **C.** Bar graph showing the mean maximum platelet aggregation in response to various doses of thrombin or collagen. # indicates a significant statistical difference (p<0.05) from non-stimulated resting human washed platelets (CTL). n = 5 different blood donors. **D–E**. DIC microscopy images of the morphological aspect of platelets after incubation with or without the indicated concentrations of thrombin (**D**) or collagen (**E**). The images shown are representative of 6 independent experiments performed with different blood donors. Scale bars = 5 µm. White arrowheads indicate platelet membrane protrusions, black arrows indicate microvesicles.

To assess platelet activation and triggering of their thrombogenic phenotype by thrombin and collagen, we first analyzed aggregation and shape changes of human washed platelets in response to increasing concentrations of these two primary platelet agonists. Aggregation of stirred platelets was induced by thrombin at the highest concentration tested (5 nM), but not at the intermediate (0.5 nM) or low concentration (0.05 nM) (Figures 1A, C). To determine individual platelet morphological response, human washed platelets were incubated with the agonists without stirring and analyzed by DIC microscopy. All three thrombin concentrations provoked obvious changes in platelet shape, with membrane protrusions being already visible at the lowest thrombin concentration (Figure 1D). In addition to intensifying the formation of membrane protrusions, increasing thrombin concentration further led to platelet swelling and emission of microvesicles (Figure 1D). Thus, at low and intermediate doses of thrombin, despite the absence of an aggregative response, platelets already showed signs of activation at the individual level.

In response to collagen, platelet aggregation occurred at both the intermediate (5 µg/mL) and high concentration (25 µg/mL), but not at the low concentration (0.25 µg/mL) (Figures 1B, C). Regarding platelet shape, collagen led to much more discrete changes than thrombin, with the sole induction of membrane protrusions at both intermediate and high concentrations, and no changes at the low concentration (Figure 1E).

### Phosphatidylserine exposure

**Figure 2:**
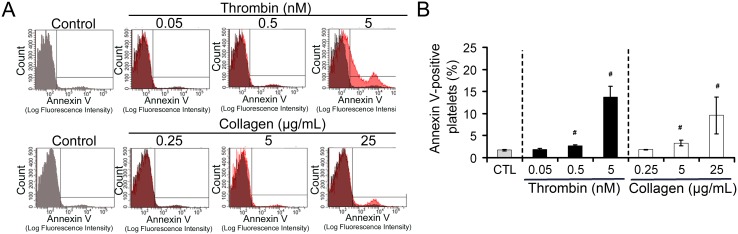
Dose-response analysis of thrombin- and collagen-induced phosphatidylserine exposure in human platelets. Phosphatidylserine exposure by human washed platelets was quantified by measurement of fluorescent annexin V binding in flow cytometry. **A**. Representative flow cytometry histograms of annexin V binding to non-stimulated resting (control) platelets or to platelets incubated with various concentration of thrombin or collagen, as indicated above the charts. The histogram obtained for control platelets was superimposed in grey to that of stimulated platelets for better visualization of fluorescence shifts. **B**. Bar graph representing the mean annexin V binding to non-treated control platelets (CTL) and to thrombin- or collagen-stimulated platelets. Results are expressed as percent relative to the mean fluorescence intensity of CTL platelets. n = 4 different blood donors, # indicates a significant statistical difference (p<0.05) from CTL platelets.

Clot formation does not only rely on the adhesive and aggregative properties of activated platelets but also on their ability to support coagulation, notably through the exposure of phosphatidylserine at their surface [24]. Therefore, to further evaluate the thrombogenic phenotype and activity of human platelets exposed to various concentrations of collagen or thrombin, we compared their level of surface phosphatidylserine to that of resting platelets. Annexin V binding to platelets, a well established marker of phosphatidylserine surface exposure [25], was not modified by low (0.05 nM) concentration of thrombin (Figures 2A, B). However, at the highest concentration of thrombin (5 nM), which caused platelet aggregation (Figures 1A, C) and important shape changes (Figure 1D), a significant increase in annexin V binding to platelets was observed, thus indicating the induction of phosphatidylserine externalisation (Figures 2A, B). A modest but statistically significant increase in annexin V binding was also observed in response to the intermediate dose of thrombin (0.5 nM). Regarding collagen-stimulated platelets, annexin V binding to platelet was also observed at concentrations inducing aggregation (5 and 25 µg/mL) but not at 0.25 µg/mL (Figures 2A, B).

### P-selectin surface expression

**Figure 3:**
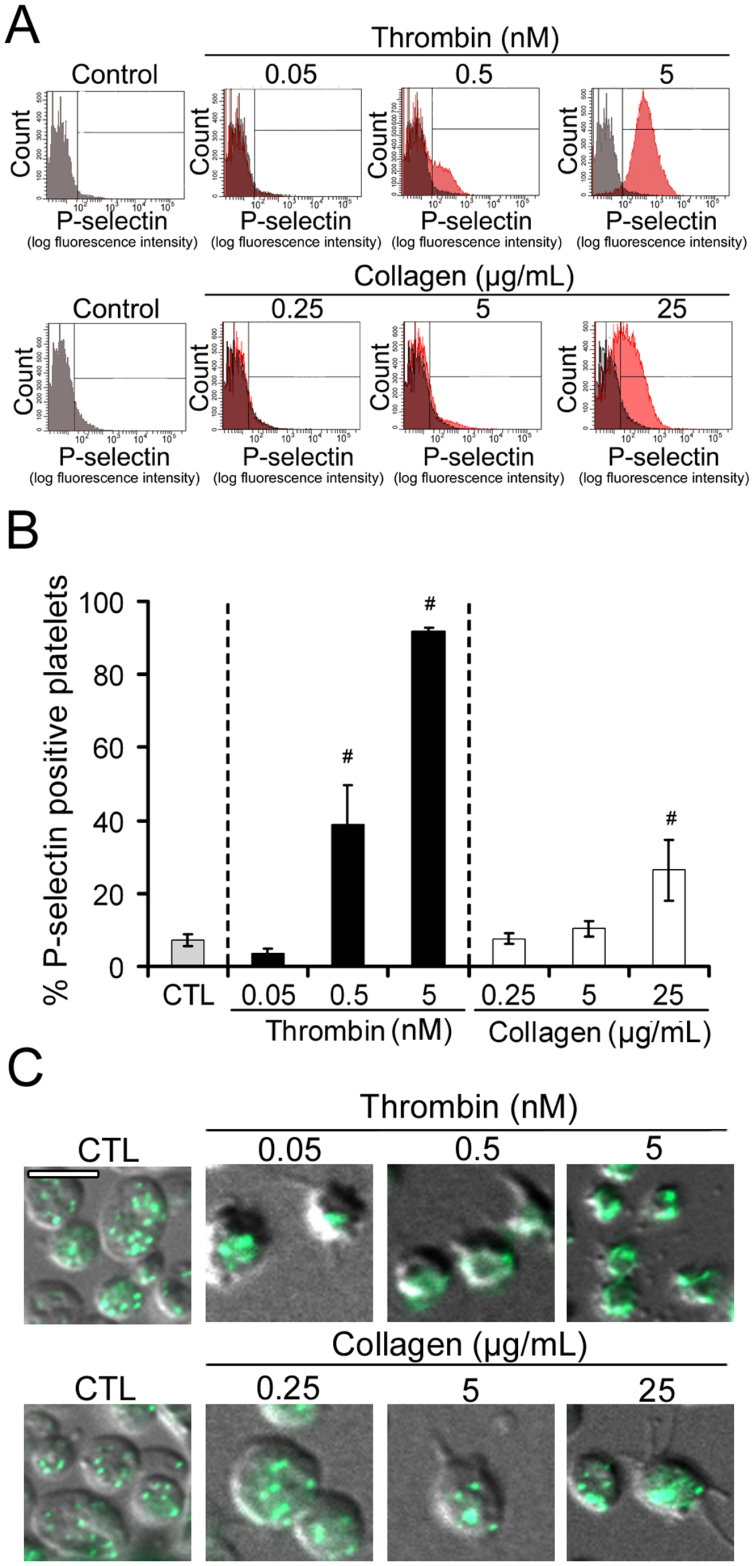
Dose-response analysis of thrombin- and collagen-induced P-selectin surface expression. **A**. Representative flow cytometry histograms of P-selectin surface expression of non-treated (control) and thrombin- or collagen-treated platelets, as indicated. The histogram obtained for control platelets was superimposed in grey to that of stimulated platelets for better visualization of fluorescence shifts. **B**. Bar graph representing the mean percentage of P-selectin-positive cells of non-treated control (CTL) and thrombin- or collagen-stimulated platelets. n = 8 independent experiments with different blood donors, # indicates a significant statistical difference (p<0.05) from CTL. **C**. Immunolocalization of P-selectin (green) in permeabilized control and agonist-stimulated platelets. Merged images of DIC and green fluorescence are shown. Bar = 5 µm.

P-selectin, a transmembrane protein anchored in the membrane of platelet alpha-granules, is translocated to the platelet surface during activation-associated alpha-granule secretion [26], [27]. For this reason, measurement of P-selectin surface expression by flow cytometry is widely used as a marker of platelet activation and degranulation, and this, both in clinical and experimental settings [28]–[30]. When assessing platelet activation status and alpha-granule secretion by this method, we observed that P-selectin surface expression was induced by thrombin at the highest dose of 5 nM, and also, though much more slightly, at the intermediate dose of 0.5 nM (Figures 3A–B). Collagen significantly increased P-selectin exposure only at the highest dose (25 µg/mL) (Figures 3A–B). These results were confirmed by immunostaining. A granule-like punctiform staining was observed on control resting platelets (Figure 3C). The same punctiform staining was obtained for platelets treated with 0.25 and 5 µg/mL collagen (Figure 3C). In contrast, platelets that had been activated by 5 nM thrombin showed a clear peripheral distribution of P-selectin. An “intermediate” staining pattern was observed for platelets activated by 0.5 nM thrombin or 25 µg/mL collagen, with a more diffuse staining and a loss of granular pattern (Figure 3C).

### Secretion of dense and alpha granule-derived bioactive agents

**Figure 4:**
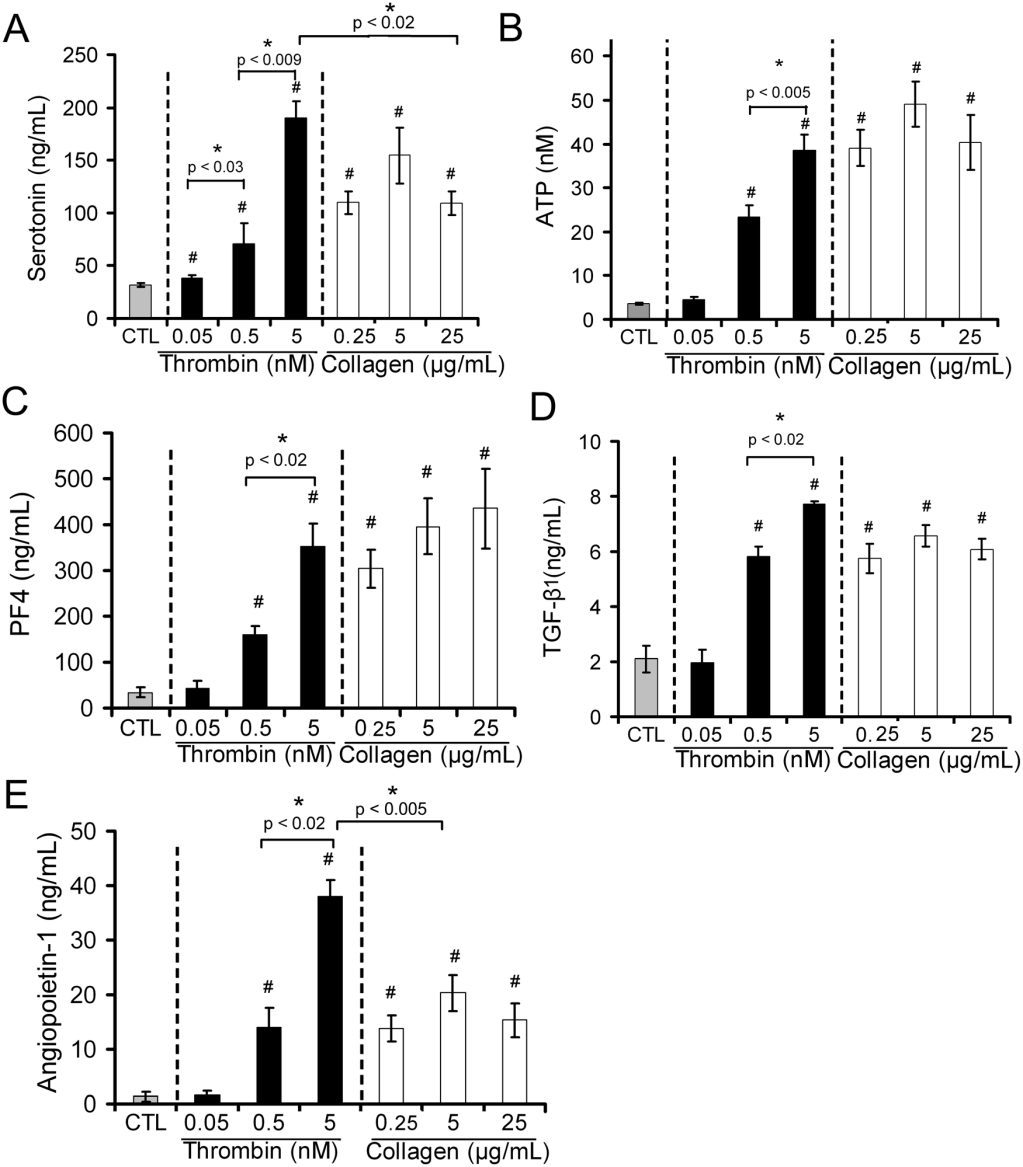
Dose-response relationship between agonist concentration and secretion of dense and alpha-granule-derived bioactive agents. The release of dense and alpha-granule components by human platelets in response to various concentrations of thrombin and collagen was assessed and compared by measuring serotonin (**A**), ATP (**B**), platelet factor 4 (PF4) (**C**), TGF-β1 (**D**), and angiopoietin-1 (**E**) levels in platelet supernatants. All bar graphs represent the mean levels calculated from 6 independent experiments performed with different blood donors. # indicates a significant statistical difference (p<0.05) from CTL.

In addition to provoking platelet shape change, aggregation and procoagulant activity, platelet activation is also known to induce the secretion of a multitude of soluble bioactive agents from platelet storage granules. Therefore, in order to further characterize the activation status of platelets exposed to various doses of thrombin and collagen, we measured and compared the release of soluble factors by control and agonist-stimulated platelets. Supernatants recovered from the very same platelet samples that were analyzed in flow cytometry and in DIC and fluorescence microscopy were then analyzed for the presence of platelet-derived soluble factors. For this analysis, we focused on factors for which platelets represent the main source in blood: serotonin and ATP, which are stored in platelet dense granules [19], [31], and PF4, TGF-β1, and angiopoietin-1, all of which are abundantly stored in platelet alpha-granules [19], [32]–[35]. The release of serotonin by platelets was increased in a dose-dependent manner by thrombin with statistical significance being reached already at the lowest concentration tested (0.05 nM) (Figure 4A). In contrast, there was no real dose–response effect for collagen. In fact, collagen at the lowest dose (0.25 µg/mL) significantly and substantially stimulated serotonin secretion with no notable further increase at higher doses (Figure 4A). Notably, the response profiles of platelets to thrombin and collagen were confirmed when dense granule secretion was assessed by measurement of ATP levels (Figure 4B). For PF4, TGF-β1, and angiopoietin-1, stimulation of platelets with thrombin and collagen led to secretion patterns comparable to those obtained for serotonin (Figures 4A–E), with a clear dose-response effect for thrombin and an almost maximum effect of collagen from the lowest dose used.

**Figure 5:**
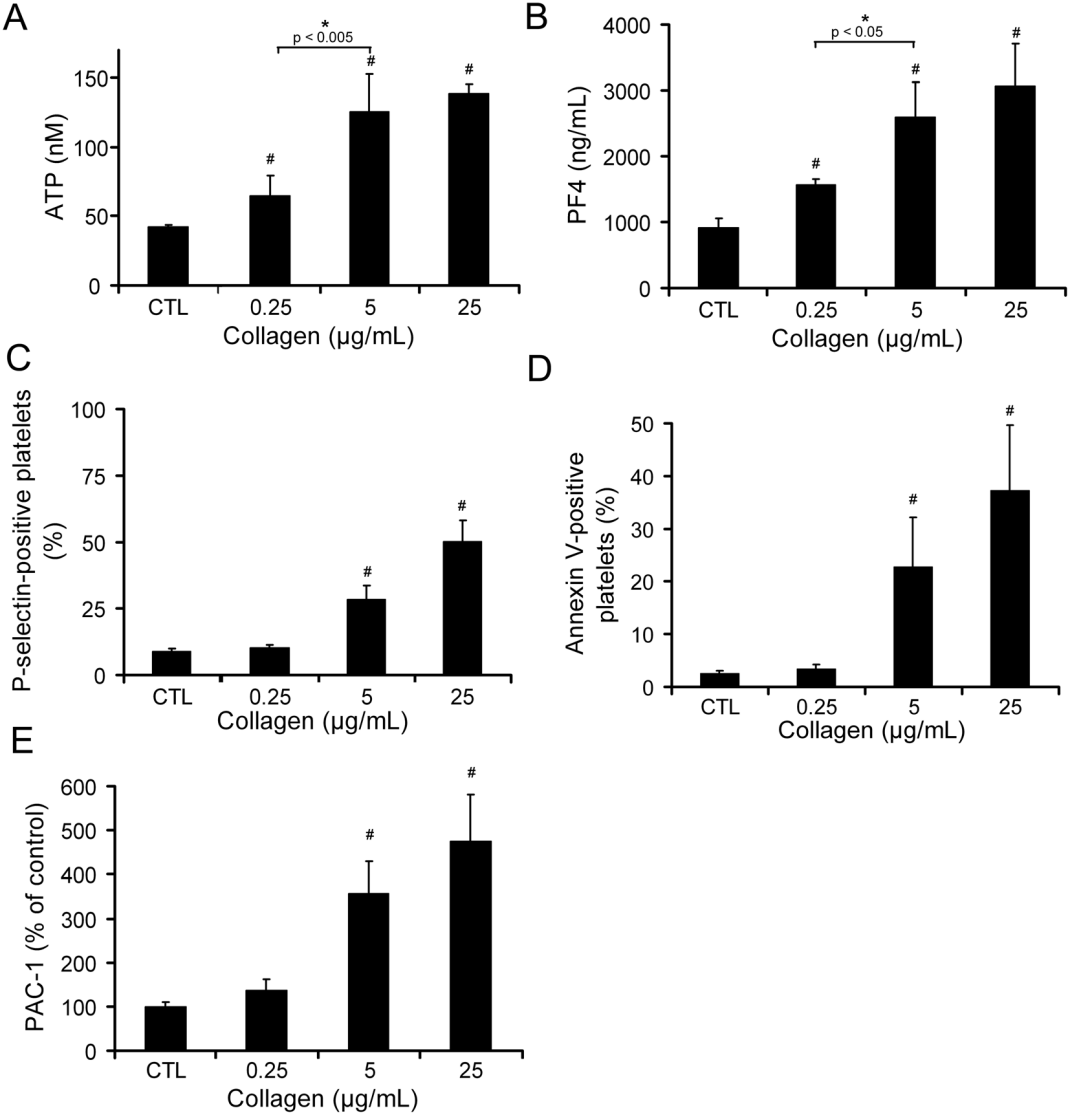
Comparison of soluble factor release and surface activation marker expression by stirred platelets exposed to collagen. **A**. ATP levels in suspensions of stirred platelets as determined using a luminescence-based assay. **B**. PF4 levels in suspensions of stirred platelets as determined using a fluorescent microspheres-based flow cytometric immunoassay. **C–E**. Surface exposure of P-selectin (**C**) and phosphatidylserine (**D**), as well as binding of the PAC-1 antibody to activated GPIIb/IIIa (**E**) on stirred platelets were measured by flow cytometry. All bar graphs represent means calculated from a minimum of 4 independent experiments performed with different blood donors. # indicates a significant statistical difference (p<0.05) from CTL.

Taken together, these results suggest that collagen at low dose can efficiently induce the release of dense- and alpha granule-derived soluble factors without triggering aggregation (Figure 1B), phosphatidylserine exposure (Figure 2), or P-selectin surface expression (Figure 3). However, because centrifugations were required for the preparation of platelet supernatants while all other parameters were measured in the absence or before centrifugation, we investigated whether the release of soluble factors by platelets in response to low-dose collagen in the absence of any other notable changes could be linked to this technical issue. To recover platelet releasates without the need for centrifugation, experiments using transwell systems (0.4 µm pore size) were performed. Platelets were placed and stimulated into the upper chamber (transwell insert) and, at the end of the incubation, the cell-free medium containing diffusible secretion products was directly collected from the lower chamber while platelets were recovered from the upper chamber for flow cytometric analysis. In these conditions, the results from flow cytometry analysis of P-selectin and phosphatidylserine expression were identical to those presented in Figure 2 and 3 (and therefore not shown). As for the release of soluble factors, while the response pattern of platelets to thrombin remained unchanged (not shown), that to collagen, on the other hand, was appreciably modified. Indeed, although in these conditions collagen did induce a statistically significant increase in the release of dense and alpha granule-derived soluble factors from the lowest concentration tested (0.25 µg/mL), a dose-dependent profile was observed (Figure S1), contrasting with the maximal effect already obtained at the lowest collagen dose when analysing centrifugation supernatants (Figures 4A–D). This dose-dependent profile was further confirmed by experiments in which platelets were stimulated under stirring conditions. In these experiments, the release of soluble factors from dense and alpha granules was assessed directly in platelet suspensions. Dense granule secretion was estimated by measuring ATP levels using a luminescence-based assay and for quantification of alpha granule secretion, we developed a microspheres*-*based flow cytometric immunoassay. Stimulation of stirred platelets with collagen induced a dose-dependent increase in the release of ATP and PF4, with statistical significance being reached from the lowest collagen dose (Figure 5A–B). Again, the release of these soluble factors in response to 0.25 µg/mL collagen occurred in the absence of significant P-selectin or phosphatidylserine surface exposure, and of integrin activation as assessed by PAC-1 binding to platelets (Figure 5C–E). Such a secretory response was not obtained for collagen at 0.125 µg/mL (not shown). Notably, and in contrast to what was observed on platelets stimulated in static conditions (Figure 2), a significant increase in P-selectin surface expression was induced by collagen at 0.5 µg/mL (Figure 5C), a concentration that induced platelet aggregation (Figure 1). Taken together, these results show that collagen at low dose can efficiently induce the release of dense- and alpha granule-derived soluble factors without triggering aggregation, phosphatidylserine exposure, or P-selectin surface expression. They further show that this secretory response is exaggerated by centrifugation of platelets.

### Role of calcium and GPVI signaling in low-dose collagen-induced platelet secretion

**Figure 6:**
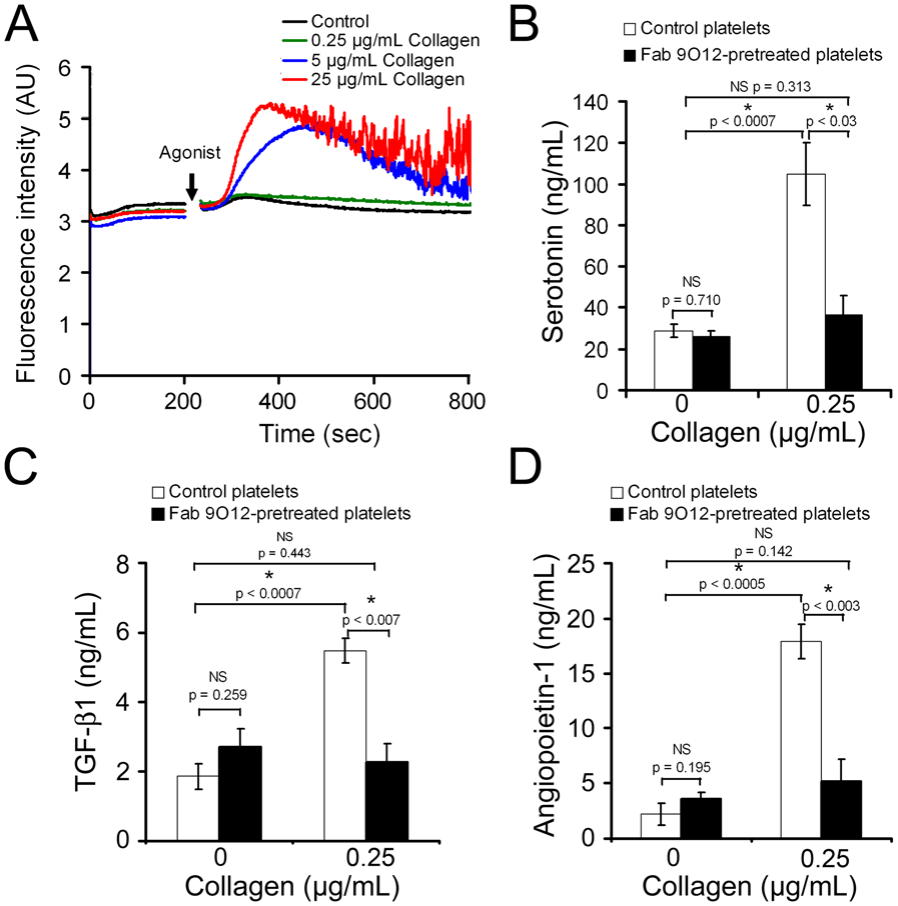
Low dose collagen-induced platelet secretion requires GPVI signalling. **A.** Intracellular calcium levels in collagen-stimulated platelets as assessed by measurement of calcium-dependent fluorescence of Oregon Green 488 BAPTA-1AM-loaded platelets. The calcium curves shown are representative of four independent experiments using different blood donors. **B–D**. To assess the contribution of GPVI-signalling to low-dose collagen-induced secretion of platelet soluble factors, platelets were stimulated with 0.25 µg/mL collagen in the presence or absence of a GPVI blocking Fab fragment antibody (Fab 9O12, 50 µg/mL) and the levels of serotonin (**B**), TGF-β1 (**C**), and angiopoietin-1 (**D**) were then measured in platelet supernatants. All bar graphs represent the mean levels from a minimum of 4 independent experiments. NS: non-statistically significant.

Activation of platelets by most stimulatory agents leads to a rise in the concentration of cytosolic Ca^2+^ which can elicit various platelet responses including shape change, spreading, adhesion, aggregation, procoagulant activity, and granule secretion [36], [37]. Therefore, we verified whether or not stimulation of platelets with low-dose collagen (0.25 µg/mL), which led to secretion of platelet soluble factors in the absence of other notable changes (Figures 1, 2, 3, 4, 5), was associated with an increase in cytosolic Ca^2+^. Collagen at intermediate and high concentration triggered an initial continuous rise followed by oscillations in intraplatelet Ca^2+^ (Figure 6A) while thrombin at intermediate and high concentration provoked a steep and transient calcium peak (Figure S2). In contrast, no change in intraplatelet Ca^2+^ levels occurred after addition of collagen at 0.25 µg/mL (Figure 6A). Similar absence of calcium was also observed when stimulating platelets with 0.05 nM thrombin (Figure S2), a concentration for which the only notable changes observed were a slight shape change and a discrete release of serotonin (Figures 1, 2, 3, 4, 5). These results show that low-dose collagen-induced platelet activation is not associated with a substantial rise in intracellular Ca^2+^.

Since GPVI is the major collagen receptor on platelets and mediates collagen-induced platelet activation in regard to platelet aggregation and procoagulant activity, we assessed the contribution of GPVI-signalling to low-dose collagen-induced secretion of platelet soluble factors. For this, platelets were stimulated with 0.25 µg/mL collagen in the presence or absence of a GPVI blocking Fab fragment antibody (Fab 9O12, 50 µg/mL) and the levels of serotonin, TGF-β1, and angiopoietin-1 were then measured in platelet supernatants. For all three factors, the secretory response of platelets to low-dose collagen was completely inhibited by blocking of GPVI (Figure 6B–D), thus demonstrating its dependency on GPVI signalling.

## Discussion

In the present study, we investigated whether or not platelets could display state(s) of activation different from that known to support their role in primary hemostasis and thrombosis. To answer this question, we analyzed and compared the changes in platelet phenotype caused by various doses of thrombin and collagen which represent the two strongest physiological primary agonists of platelets. We found that collagen at low dose (0.25 µg/mL) selectively triggers a platelet secretory response without causing any of the other major changes that usually accompany platelet activation and that were observed at higher collagen concentrations. More precisely, stimulation of platelets with low-dose collagen led to significant secretion of dense- and alpha granule-derived soluble factors, and this without causing platelet aggregation or surface exposure of phosphatidylserine and P-selectin. Notably, such a selective secretory response of platelets was not observed when thrombin was used as an agonist. In fact, thrombin at low dose only slightly increased serotonin secretion by platelets and did not lead to secretion of alpha granule-derived PF4, TGF-β1, or angiopoietin-1. Furthermore, this secretory response was associated with a clear induction of platelet shape change. Although at higher concentrations thrombin did induce a significant release of both dense and alpha granule-derived soluble factors, this response was associated with one or more additional platelet activation-associated changes. Taken together, these results show that as previously described for P-selectin and phosphatidylserine exposure [25], [38], selective triggering of the platelet secretory response is dependent on both the agonist used and its concentration. In addition to support the idea that the response pattern of activated platelets is context-related, our data indicates that platelet activation includes situations in which the secretory activity of platelets is dissociated from their ability to form thrombi.

Interestingly, we found that the release of various alpha granule-derived soluble factors by platelets exposed to low-dose collagen was not associated with an increase in the surface expression of P-selectin, a transmembrane protein widely known to reside in the alpha granule membrane. This discrepancy between the secretion pattern of membrane P-selectin and alpha granule-derived soluble factors might have several implications. First, it might reflect differences in the kinetics of secretion between soluble and transmembrane factors that would however be released from similar granules. Results from a recent study by Jonnalagadda *et al.* have shown that molecules stored in presumably similar granules could display differences in their rate of secretion due to possible differences in solubility, granule shape, and/or granule-plasma membrane fusion routes [39]. One can then imagine that it might be easier for a soluble factor to diffuse out of the open canalicular system (OCS) into the extracellular medium than for a transmembrane protein to migrate from the OCS to the surface within the membrane lipid bilayer. Moreover, immunolocalisation of P-selectin in low-dose collagen-treated platelets revealed a granular staining pattern similar to that of untreated platelets. Therefore, the absence of surface P-selectin on low-dose collagen-stimulated platelets might just reflect the need for platelet contraction and shape change for P-selectin to be externalized together with the OCS. Also, another possibility is the existence of various subtypes of and/or subdomains in alpha granules whose cargo molecule secretion would be differentially regulated in a dose- and agonist-dependent manner. Several studies have shown that differential sorting of alpha granule cargo in distinct subtypes or domains of alpha granules can result in their differential release [10], [40]–[43]. In this context, it is noteworthy that the maximum effect of thrombin on serotonin and angiopoietin-1 secretion was higher than that of collagen, showing that thrombin can specifically increase the secretion of these factors. This could be achieved by complete versus partial emptying of similar alpha granules, or by mobilization of additional and/or different alpha granules by thrombin as compared to collagen. All in all, these differences in the release of diffusible factors in response to various stimuli that all cause maximal aggregation reinforces the idea that platelet activation is far from being univocal [8], [39].

Considering the fact that platelets have been shown to exert thrombus-independent regulatory actions in various inflamed organs and solid tumors [18], [19], [44], our findings bring new mechanistic insights that might partly explain these effects. Previous studies have suggested the implication of platelet granule content in the prevention of tumor hemorrhage by platelets, possibly through regulation of vascular permeability and immune cell activities [19], [44], [45]. Also, platelet-derived serotonin has been shown to mediate liver regeneration [46]. We show here that collagen at low dose leads platelets to release serotonin, PF4, angiopoietin-1, and TGF-β1, all of which have immunomodulatory properties and/or the ability to regulate endothelial permeability. Moreover, it was shown recently that the vasculoprotective action of platelets in inflamed organs is dependent on ITAM receptors including GPVI [21], a receptor which we found to mediate the secretory response of platelets to low-dose collagen.

Furthermore, we observed that this selective secretory response of platelets to low-dose collagen was not associated with a sustained rise in intracellular calcium. In a similar manner, the prevention of inflammatory bleeding by platelets was shown previously to be independent of the calcium and diacylglycerol-regulated guanine nucleotide exchange factor 1 (CalDAG-GEF1) [18], [19], [21], a molecule which is central to calcium signalling in platelets [47]. Whether or not the collagen-induced platelet secretory phenotype we described here does actually contribute to the prevention of inflammatory bleeding by platelets however remains to be verified. The absence of substantial rise in intracellular calcium might also indicate that basal calcium levels are sufficient to promote the secretory response induced by low-dose collagen. Studies of secretory cells such as adrenal chromaffin cells and neurons have shown that secretion can occur at low intracellular calcium levels through a mechanism of incomplete and transient fusion of secretory vesicles with the plasma membrane. The shift from this so-called “kiss-and-run” mechanism to full exocytosis occurs when cell stimulation is accompanied by high intracellular calcium levels [48], [49]. A kiss-and-run mechanism, for which evidence of its existence in platelets has been brought by single-cell amperometry studies [50], [51], could thus explain the secretory response of platelets to low-dose collagen while full collapse fusion with externalization of P-selectin would occur at higher agonist concentrations associated with a marked increase in intracellular calcium.

In conclusion, our results show that platelet activation goes beyond the mechanisms leading to thrombus formation and also includes alternative platelet phenotypes that might contribute to their thrombus-independent functions. Also, the fact that GPVI can mediate the release of potent immunomodulators and regulators of vascular permeability without evoking platelet aggregation or procoagulant activity provides a new conceptual view on GPVI/collagen interactions that reconciles GPVI with its immune receptor nature.

## Supporting Information

Figure S1 **Secretory response of platelets to collagen in static conditions.** To assess the release of soluble factors by platelets in response to collagen independently of mechanical agitation, platelets were placed and stimulated into transwell inserts (pore diameter 0.4 µm), and the cell-free medium containing diffusible secretion products was collected from the lower chamber. Levels of serotonin (**A**), ATP (**B**), and platelet factor 4 (PF4) (C) in platelet releasates were measured as reflects of dense and alpha granule secretion, respectively. Results are expressed as percent relative to the mean levels found in releasates of unstimulated control platelets. n  =  at least 6 different blood donors, # indicates a significant statistical difference (p<0.05) from unstimulated control platelets.
(TIF)

Figure S2 **Intracellular calcium levels in thrombin-stimulated platelets.** Calcium levels of Oregon Green 488 BAPTA-1AM-loaded platelets treated with thrombin were measured using a fluorescence microplate reader. The calcium curves shown are representative of four independent experiments using different blood donors.
(TIF)

Raw Data S1 (XLS)
