# Supplementary figures and images for: Collagen Can Selectively Trigger a Platelet Secretory Phenotype via Glycoprotein VI
Source: PLoS One. 2014 Aug 12;9(8):e104712. doi: 10.1371/journal.pone.0104712 (PMC4130581; doi:10.1371/journal.pone.0104712)

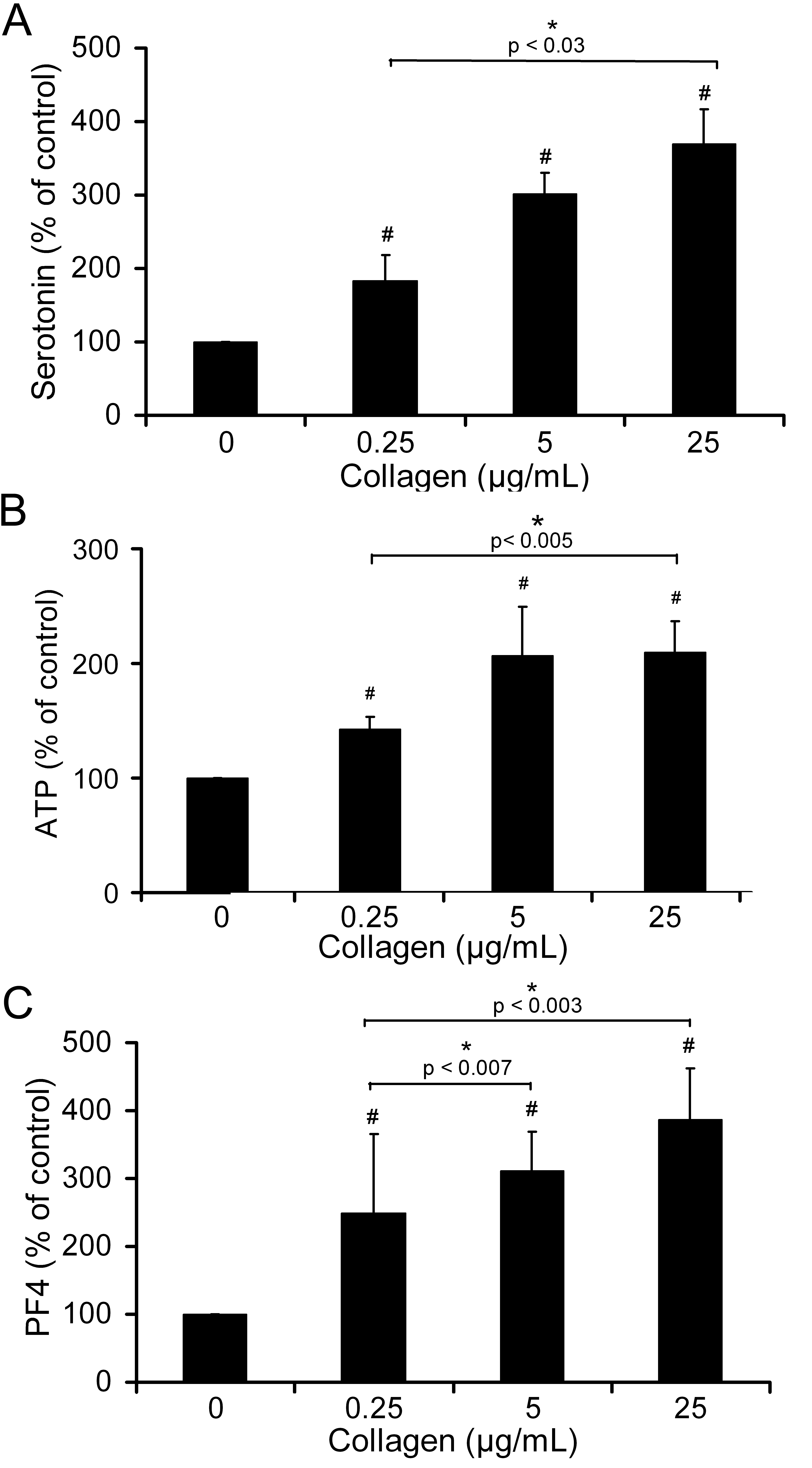

Supplement: Figure S1 — Secretory response of platelets to collagen in static conditions. To assess the release of soluble factors by platelets in response to collagen independently of mechanical agitation, platelets were placed and stimulated into transwell inserts (pore diameter 0.4 µm), and the cell-free medium containing diffusible secretion products was collected from the lower chamber. Levels of serotonin (A), ATP (B), and platelet factor 4 (PF4) (C) in platelet releasates were measured as reflects of dense and alpha granule secretion, respectively. Results are expressed as percent relative to the mean levels found in releasates of unstimulated control platelets. n = at least 6 different blood donors, # indicates a significant statistical difference (p<0.05) from unstimulated control platelets. (TIF) [file pone.0104712.s001.tif]

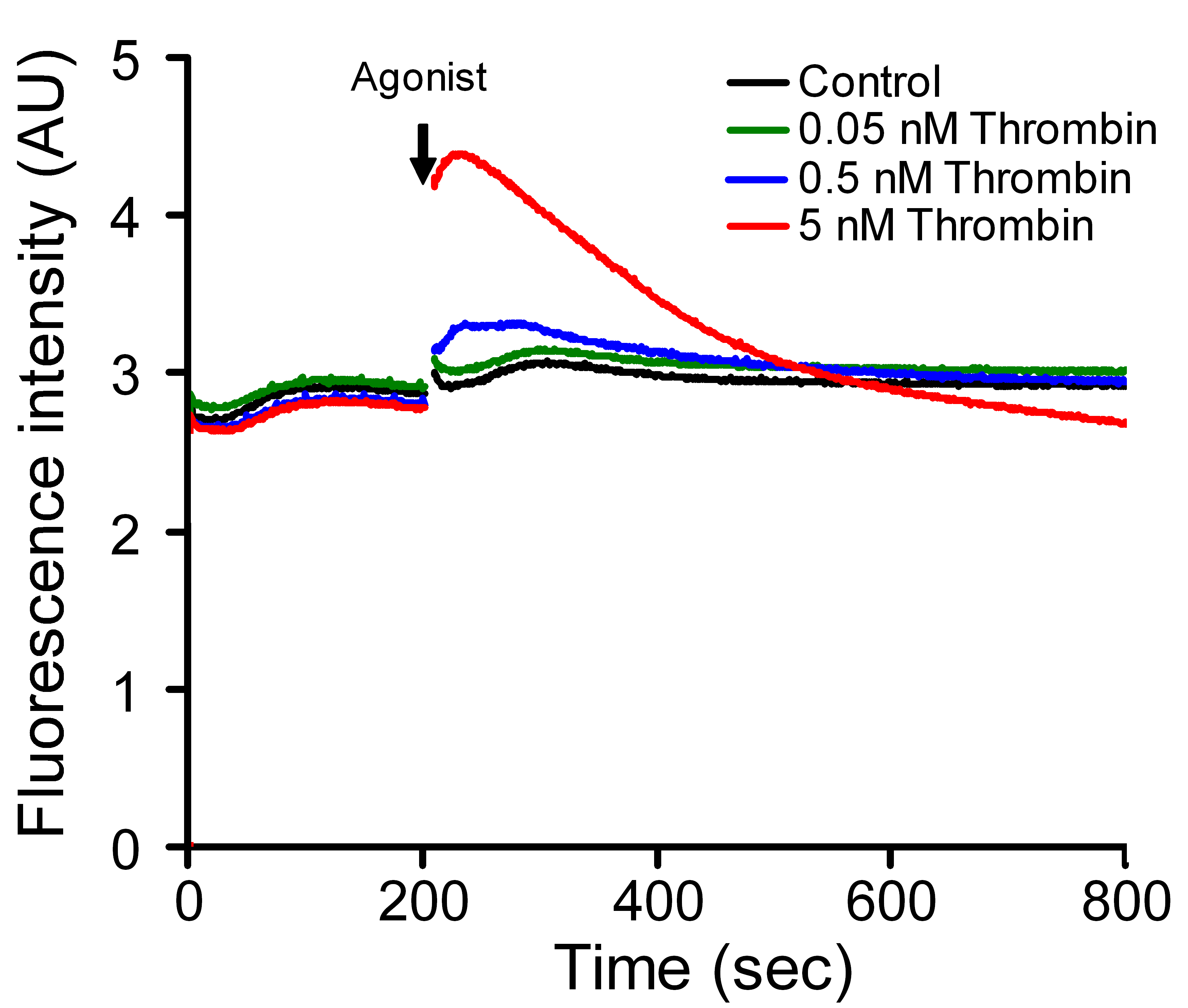

Supplement: Figure S2 — Intracellular calcium levels in thrombin-stimulated platelets. Calcium levels of Oregon Green 488 BAPTA-1AM-loaded platelets treated with thrombin were measured using a fluorescence microplate reader. The calcium curves shown are representative of four independent experiments using different blood donors. (TIF) [file pone.0104712.s002.tif]
